# The Role of Artificial Intelligence in Clinical Psychology: How AI and NLP Systems Are Reshaping Psychological Interventions. A Systematic Review

**DOI:** 10.1002/cpp.70242

**Published:** 2026-02-25

**Authors:** Luisa Orrù, Stefania Mannarini

**Affiliations:** ^1^ Department of Philosophy, Sociology, Education and Applied Psychology (FiSPPA) University of Padova Padova Italy; ^2^ Center for Intervention and Research on Family Studies (CIRF) University of Padova Padova Italy; ^3^ Language Computing and Human AI Interaction Laboratory University of Padova Padova Italy

**Keywords:** artificial intelligence, clinical psychology, digital mental health, natural language processing, psychological treatment

## Abstract

Artificial Intelligence (AI) technologies are rapidly evolving and their integration into psychological practices has progressively expanded, offering new tools for diagnosis, treatment and therapeutic monitoring. This review examines the transformative role of AI, particularly Natural Language Processing (NLP) systems, in reshaping clinical psychology and digital mental health interventions (DMHIs). In particular, it explores how AI and NLP can facilitate human‐machine interaction in therapy by analysing how language is used within clinical conversations and providing personalized, real‐time interventions. Following PRISMA guidelines, a systematic review of literature from 2019 to 2025 identified 17 studies that met inclusion criteria, emphasizing AI's use in psychological assessment and intervention. The review focuses on two key aspects: the functions and applications of NLP‐based systems in clinical practice and the advantages and benefits they offer for both psychologists and patients. Findings suggest that NLP‐driven AI systems enhance both patient engagement and clinician efficiency, offering scalable, cost‐effective solutions that improve access and personalization. However, challenges remain, including ethical concerns around data privacy, lack of standardization, limited generalizability across disorders and reduced human empathy. Moreover, current systems are primarily designed for well‐defined conditions like anxiety and depression, with limited applicability to complex or comorbid psychological presentations. This review underscores the importance of supervised, ethically governed AI implementation. While AI holds substantial promise in augmenting clinical psychology, its success depends on maintaining human oversight, ensuring transparency and establishing shared scientific and ethical standards across the psychological community.

## Summary

- Through the systematic review of the existing literature regarding the use of AI in clinical psychology, practitioners could know how AI and NLP systems are (re)shaping psychological interventions to improve them for digital mental health (DMH).
- The study describes characteristics, features and tasks of AI systems in clinical psychology, the main techniques currently adopted, from which practitioners can find information to choose the best tools for their work objectives, adapting to specific individual psychological needs.
- Practitioners could evaluate AI systems' impact on DMH promotion: beneficial effects of AI systems for patients' mental health and related challenges. Indeed, while on one hand the implementation of AI in clinical psychology has demonstrated significant improvements in psychological well‐being, on the other hand some studies have reported less conclusive findings.

## 1 Introduction

In recent years, rapid advancements in Artificial Intelligence (AI) have made it an integral part of people's everyday life. The overarching goal is to develop increasingly precise AI systems, capable of mastering a wide range of tasks such as learning, planning, reasoning and language comprehension (Stuart and Peter 2016). AI systems can be understood as integrated architectures that coordinate multiple components to handle complex tasks (Stuart and Peter 2016). They are based on a series of mathematical approaches employed to teach AI how to process data and make decisions to increase efficiency, analytics and automation capabilities (Sarker 2022).

The main techniques currently adopted include: Machine Learning (ML), which is a core subfield of AI that enables machines to learn from data and make decisions based on them without being explicitly programmed to do so (Alpaydin 2020); Deep Learning—a class of ML algorithms using multiple layers to extract features from raw inputs—which uses artificial neural networks (NNs) to analyse large amounts of complex data (Goodfellow et al. 2016); Natural Language Processing (NLP), a subfield of AI addressing human‐machine interactions, which allows for understanding, interpreting and responding to natural language (Chang et al. 2018; Jurafsky and Martin 2023). Another category of models that boosted the adoption of AI across various domains is Large Language Models (LLMs), which are designed to understand, process and generate natural language (Raiaan et al. 2024). LLMs are a class of AI systems trained on vast amounts of textual data in order to learn the distinctive features of natural language, such as structure, meaning and context, and perform several language tasks (Bommasani et al. 2022). After learning such features, they can produce new meaningful content in the form of text, images or audio (Feuerriegel et al. 2024).

Based on these AI models, increasingly sophisticated tools capable of generating content and interacting with humans have emerged. AI tools are particular applications or algorithms that perform a specific task (Stuart and Peter 2016). Notable examples include chatbots such as ChatGPT, Gemini, LLama and Bard. These are AI‐based systems that, leveraging NLP and ML techniques, can interact with users through natural language (Radziwill and Benton 2017). As AI knowledge and understanding have advanced, these tools have evolved significantly, finding applications across diverse fields such as economics, finance, engineering, education, medicine and psychology (Stone et al. 2022).

In psychology, AI was first introduced in 1966, with the development of ELIZA: the first chatbot designed to simulate psychotherapy (Adamopoulou and Moussiades 2020). Rather than providing interpretative responses, ELIZA merely rephrased users' statements through a combination of text recognition models (Pattern Matching) and predefined rules, identifying keywords and generating responses accordingly (Gwon and Seo 2021).

Since ELIZA, AI began to be utilized as a supportive tool in psychiatric diagnosis, enabled by ML and big data analysis. Nowadays, ML techniques are being developed for screening models that assess the risk of mental disorders (Shatte et al. 2019). For example, neuroimaging techniques help distinguish Alzheimer's symptoms from normal aging (Doan et al. 2017), detect vulnerability to depression (Sato et al. 2015) and assess the risk of psychosis (Koutsouleris et al. 2012). Regarding the diagnostic process, AI applications use large datasets to enable early diagnoses (Dimitriadis et al. 2018) and to improve detection for disorders with overlapping symptomatology (Bosl et al. 2017). For example, ML integrated with electroencephalogram (EEG) can differentiate autism spectrum disorders from epilepsy, while audio‐visual data models enhance diagnostic accuracy for Alzheimer (König et al. 2015), schizophrenia (Tron et al. 2016) and suicidal intent (Pestian et al. 2016). ML‐based models help distinguish ADHD (Attention Deficit Hyperactivity Disorder) from bipolar disorders and ML techniques have been used for prognosis of certain disorders. Specifically, they have been applied to predict the long‐term outcomes of conditions such as Alzheimer, depression, psychosis and PTSD (Shatte et al. 2019). Lastly, ML techniques are also applied to monitor behavioural changes linked to mental disorders and direct patients to appropriate support systems (Orrù et al. 2024), particularly for suicide risk or addiction treatments (Shatte et al. 2019).

Linked to this, online interactions' analysis has been employed to predict individuals' ability to recover from nicotine and alcohol abuse (Shatte et al. 2019). Here is where NLP is applied. Indeed, NLP tools allow the automated analysis of large volumes of textual data, helping clinicians to identify meaningful linguistic patterns, track patient progress and improve research on therapeutic processes, ultimately providing data‐driven insights for personalized treatment (Atzil‐Slonim et al. 2024; Kuo et al. 2024). NLP models that analyse textual data (Orrù et al. 2024; Turchi et al. 2024) have been used to detect suicidal intentions from transcripts of psychological sessions (Oseguera et al. 2017), schizophrenia symptoms (Strous et al. 2009) and depressive signs from social media data (Wu et al. 2012).

Both Machine Learning and NLP have contributed to the development of more sophisticated psychological tools, especially apps and chatbots (Natale 2019). In psychological intervention, chatbots are the most employed tools: they are used, for example, for suicide prevention, widely in Cognitive‐Behavioural Therapy (CBT) – a psychotherapeutic intervention focused on identifying and modifying negative thought patterns affecting emotions and behaviours (Hofmann et al. 2012; Nakao et al. 2021) – and provide support for various disorders such as anxiety, depression and PTSD (Bertl et al. 2022). They also offer anonymous access to interventions for individuals reluctant to seek professional help due to stigma or fear of judgement, ensuring immediate support beyond traditional therapist hours (Pretorius et al. 2019).

Examples of such tools include Tess, Wysa and Woebot (Fitzpatrick et al. 2017; Fulmer et al. 2018; Gionet 2018; Inkster et al. 2018), interactive agents that can detect, report and explain expressions of emotional distress. These tools not only provide clinical explanations of users' experiences but also offer tailored advice, helping patients recognize emotional patterns and develop coping strategies for anxiety and depressive symptoms (Haque and Rubya 2023). Other examples are Kognito (Rein et al. 2018), an educational platform for suicide risk prevention and the Avatar Project (Craig et al. 2018), which helps patients manage persistent auditory and visual hallucinations related to psychosis. These latter are computer‐generated avatars interacting dynamically with patients that are used in interventions targeting disorders such as psychosis, schizophrenia, depression and phobias.

Thus, the application of AI in clinical psychology aims to enhance the efficiency and accessibility of psychological services. Particularly in the field of Digital Mental Health Interventions (DMHIs), AI‐based systems have demonstrated a broad spectrum of applications, improving both therapists' practices and users' experiences (Olawade et al. 2024). Currently, these systems can be categorized into three macro‐categories: apps and bots, avatars and robots that range from basic messaging applications with instant messaging to interactive agents. However, ethical and practical concerns remain regarding the adoption of AI, particularly in the psychological field (Dwyer et al. 2018). Key issues include privacy, data security, transparency, informed consent, bias, over‐standardization and replicability, impact on practitioners, unknown long‐term effects (Chenneville et al. 2024). Risks vary across AI applications in training, evaluation and intervention. For instance, if improperly trained, Large Language Models might reinforce diagnostic stigma and fail to comply with established ethical standards in clinical psychology (Lawrence et al. 2024). Additionally, psychotherapy frameworks differ significantly from one another, making it challenging to integrate AI systems capable of providing effective responses for highly complex conditions with comorbidities (Stade et al. 2024).

A further concern is the potential dependency patients may develop on these AI‐driven tools, hindering their ability to generalize coping strategies to real‐world human interactions. Vulnerable groups, including children, the elderly and individuals with cognitive disabilities may struggle to understand AI functionalities or mistakenly assume clinical supervision is involved (Meadi et al. 2025). Additionally, AI systems may face ethical dilemmas: for instance, detecting a high suicide risk without the ability to evaluate broader contextual clues, which are crucial in human decision‐making.

Despite these issues, AI applications in digital mental health (DMH) are expanding, given their potential for reliability, effectiveness and efficiency in clinical practice. In this context, reviewing AI integration in clinical psychology is essential to understanding both its benefits and limitations (Lee and Ahn 2020).

Considering all of the above, this study aims to provide an overview of studies examining the use of AI, with particular focus on NLP‐based systems, in the field of clinical psychology. The reason that grounded the choice to focus our investigation solely on these was that psychotherapy and clinical sessions are characterized by the patient‐practitioner interaction through question, answer and discourses, thus concerning what is continuously being said through natural language. Specifically, the review focuses on two key aspects: (1) the (functions and) applications of NLP‐based systems in clinical practice; (2) the advantages and benefits they offer for both psychologists and patients.

The review is structured as follows: the ‘Methods and Procedures’ section outlines the systematic review methodology, detailing the research process and criteria for study selection. The “Results” section presents findings from the eligible studies divided into two paragraphs, respectively (1) characteristics and tasks of AI systems; (2) beneficial effects of these systems on patients and practitioners, also highlighting the clinical pictures and interventions. Lastly, the ‘Discussion’ section synthesizes and critically discusses the main findings including ethical issues associated with AI adoption in clinical settings. It also provides an informed perspective on the future implementation of AI and NLP in psychological practice.

## 2 Methods and Procedures

### 2.1 Search and Selection Strategy

This review was conducted in accordance with the PRISMA guidelines (Preferred Reporting Items for Systematic reviews and Meta‐Analysis).

A systematic search was performed using the PsycNet, Web of Science and Scopus databases. The search query by keywords is shown in Table 1; it was the same for all three databases. Articles were automatically extracted from all databases on 8 April 2025.

**TABLE 1:** Relevant keywords for articles selection.

‘artificial intelligence’, OR ‘machine learning’	AND	‘change’, OR ‘clinical psychology’, OR ‘mental health’, OR ‘psychotherapy’, OR ‘cognitive assistant’, OR ‘psychologist’

These keywords were selected to align with the dual objective of this review: (1) to examine the applications of AI systems in the field of clinical psychology; (2) to evaluate their impact on DMH promotion and positive effects for patients and therapists.

In addition, the following filters were applied in the search:

- Publication year: > 2018 and < 2025;
- Article type: only research papers (no reviews, commentary, letters nor conference papers);
- Language: English and Italian;
- Publishing stage: only already published papers in peer‐reviewed journals (no pre‐prints, grey literature, etc.).

Subject area: Psychology, Computer Science. It was decided to search articles published from 2019 mainly due to the recency of the topic, for which it was evaluated as adequate to consider papers non‐older than 5 years. Moreover, the year before (2018) was pivotal for the developments in that field. Indeed, BERT—one of the most known Transformer models—was developed in 2018 and in 2019 Google began to use it to compute research queries. Also, in 2018 the OpenAI GPT transformer series became the state‐of‐the‐art in natural language generation (NLG). Thus, selecting papers published from the following year (2019) was considered strategic for the aim of the review.

The data collection process began at the end of November 2024 and concluded in April 2025. All retrieved studies were compiled into a unified dataset and duplicate entries were removed. Following this first screening phase, a total of 883 papers were identified.

The second screening involved evaluating the relevance of title and abstract concerning the topics of this review. The titles and abstracts of the identified studies were independently screened by two reviewers, in order to determine their relevance: the reviewers were not blinded to each other's decisions but discussed differences openly, resolving disagreements through discussion. If consensus was not reached, a third reviewer was involved to make the final decision. Decisions regarding study inclusion were recorded in an Excel spreadsheet, where each entry included the study reference, reasons for inclusion/exclusion and reviewer comments. This phase resulted in 165 selected papers.

At this point, the focus was narrowed exclusively to those addressing text analysis and NLP applications, reducing the count to 34 papers that were fully analysed. This last criterion allowed us to focus on research that delves deeper into DMH promotion in clinical psychology through the study of conversations about psychological treatments between patients and practitioners. Indeed, within the clinical psychology field, it is paramount to have access to the patients' text (their narrative) in order to be able to carry out the anamnesis, diagnosis and intervene on the reported symptoms and health configuration. The risk of bias and quality of the included studies were assessed by two independent reviewers, with a third reviewer available to resolve any disagreements and to assess internal validity. The assessment has been done at outcome level, evaluating the relevance and coherence of the data to the review questions, completeness of outcome data and handling of missing data. The results of the risk of bias assessment have been considered for the interpretation of findings, discussing studies with high risk of bias will be discussed with caution.

**FIGURE 1:**
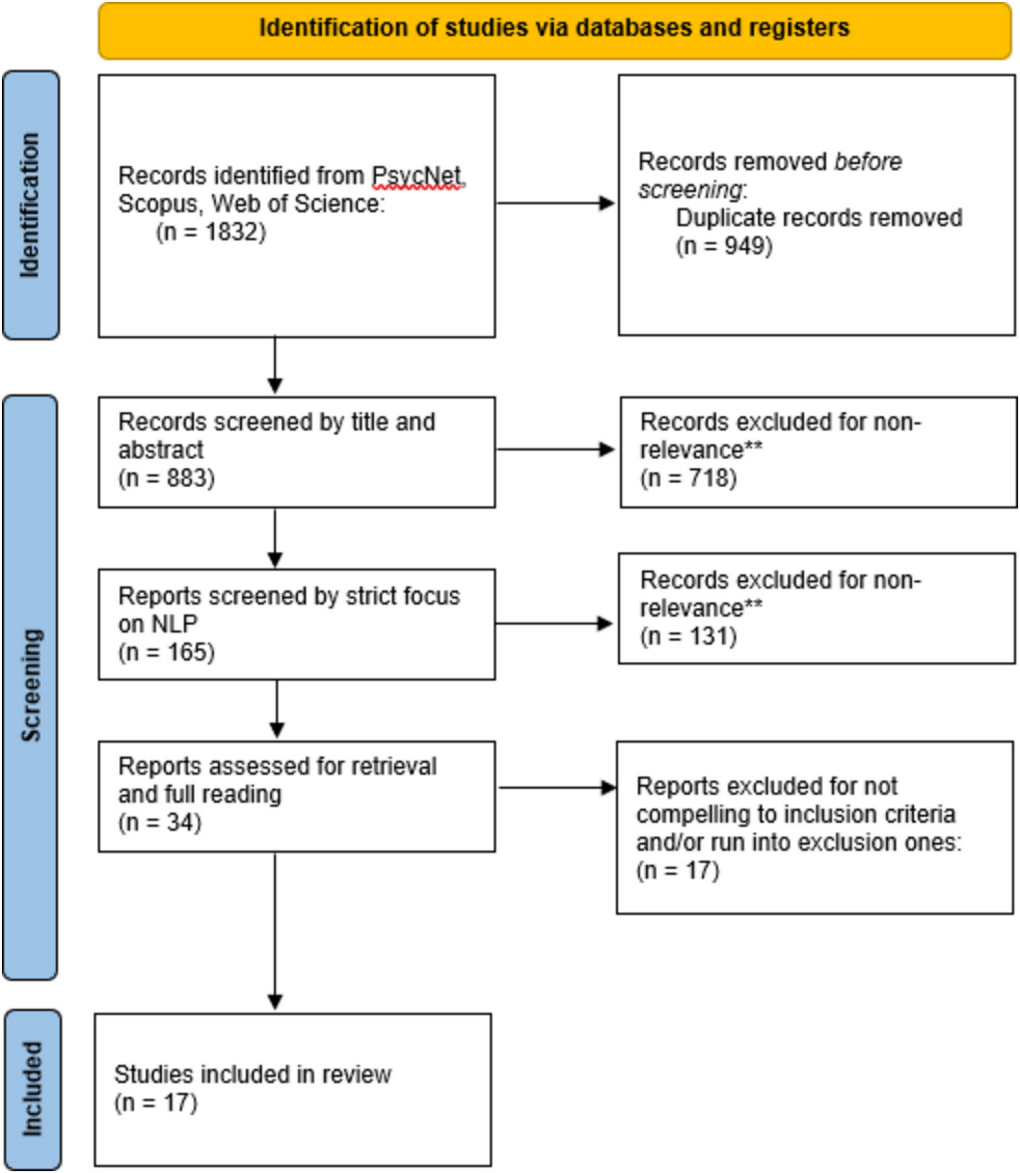
PRISMA review flowchart.

During this in‐depth analysis, the following additional inclusion criteria were applied: (3) psychological assessment or intervention as the primary focus of the paper, (4) AI used for supporting and/or enhancing interventions and (5) discussion of psychological/clinical impact of AI‐assisted intervention. Furthermore, the exclusion criteria were defined as follows: (6) Studies not related to human psychological conditions.and (7) studies describing theoretical protocols without application results. These further criteria were applied in order to guarantee relevance to the review's aims and for the field of application: indeed, as an example, maintaining studies without application results would not provide fruitful insights for clinical psychologists. Again, this process was conducted by two independent reviewers as described above. It is specified that, for both phases, inter‐rater reliability was not assessed. They extracted the data from the selected studies using a standardized data extraction form, based on the two review questions (stated above). Thus, information will relate to: name and/or type of AI system; psychological theories, methods and techniques on which the system is based; technology on which the system is based; the presence or absence of effects on patients and practitioners; beneficial effects on patients and practitioners. Ultimately, 17 papers met the full eligibility criteria and were included in this review. Figure 1 summarizes all the steps now described, from identification to screening and inclusion.

## 3 Results

### 3.1 Characteristics of Included Studies

Table 2 reports the 17 studies included in this review. The table shows some bibliographic details as well as the positioning of them in relation to:

- main target of the system (patients or therapists)
- type of intervention (supportive or substitutional)
- type of disorder (psychological or psychological on medical‐base) and sample
- psychological theory of reference

**TABLE 2:** Selected studies with bibliographic details and main characteristics (see Appendix S1 for the explanation of the categorization used to classify systems).

Title	Authors	Year	Journal	Main target of the system		Type of intervention		Type of disorder	
				Patients	Therapists	Supportive	Substitutional	Psychological	Psychological on medical‐base
Demographic and clinical characteristics associated with anxiety and depressive symptom outcomes in users of a digital mental health intervention incorporating a relational agent	Chiauzzi, E., Williams, A., Mariano, T. Y., Pajarito, S., Robinson, A., Kirvin‐Quamme, A. and Forman‐Hoffman, V.	2024	BMC Psychiatry	Woebot for Mood and Anxiety (W‐MA‐02), a relational agent that utilizes thoughtful conversational design and some NLP to deliver intervention.			Delivers intervention following CBT, IPT and DBT through a text‐based interface on a smartphone app.	Depression, Anxiety.256 participants, of which: 111 with elevated baseline levels of depressive symptoms; 107 with elevated baseline levels of anxiety symptoms.	
Computational psychotherapy system for mental health prediction and behaviour change with a conversational agent	Kolenik, T., Schiepek, G. and Gams, M.	2024	Neuropsychiatric Disease and Treatment	Novel artificial cognitive architecture that, from real‐time free text, understands its user and offers effective personalized help for relieving SAD symptoms.		LLMs generate linguistic outputs as a response to the input text in the form of motivational messages personalized to the user's personality. At the end of a specific conversation the system re‐evaluates the user's well‐being and (a) offers adapted strategies if it has not changed or (b) teach new strategies for future use if it has improved.		SAD (Stress, Anxiety and Depression)	
Future of ADHD Care: Evaluating the Efficacy of ChatGPT in Therapy Enhancement	Berrezueta‐Guzman, S., Kandil, M., Martín‐Ruiz, M. L., Pau de la Cruz, I. and Krusche, S.	2023	Healthcare	Developed a Custom ChatGPT (to be validated by therapeutic experts before being implemented) for a robotic assistant supporting ADHD therapies.			In the interaction between ChatGPT and the therapists three categories have been measured: (1) Insight into Patient's Emotional State; (2) Tailored and Personalized Responses; (3) Overall Effectiveness as a Therapeutic Tool.		ADHDSample of 10 esteemed experts in therapies for children with ADHD.
Improving the Well‐being of Adolescents With Type 1 Diabetes During the COVID‐19 Pandemic: Qualitative Study Exploring Acceptability and Clinical Usability of a Self‐compassion Chatbot	Boggiss, A., Consedine, N., Hopkins, S., Silvester, C., Jefferies, C., Hofman, P. and Serlachius, A.	2023	JMIR diabetes	COMPASS, a chatbot app intervention for adolescents T1D. Examines acceptability and potential clinical utility in adolescents with T1D and their diabetes health care professionals.		The COMPASS chatbot is designed to deliver daily content in 14 conversational lessons daily across 2 weeks, aimed at facilitating self‐compassion coping skills for adolescents with T1D.			Type 1 Diabetes (T1D). 15 to 20 adolescents with T1D (aged between 12 and 16 years) and 10 to 15 diabetes health care professionals.
Artificial Intelligence Enabled Mobile Chatbot Psychologist using AIML and Cognitive Behavioural Therapy	Omarov, B., Zhumanov, Z., Gumar, A. and Kuntunova, L.	2023	International Journal of Advanced Computer Science and Applications	AI‐enabled mobile chatbot psychologist that leverages AIML and CBT to offer personalized psychological interventions. It adapts to user's needs, recognizing their emotional state and providing personalized support accordingly.			Alternative to traditional therapy for accessible, cost‐effective and efficient mental health care solutions. It provides personalized psychological support through a mobile platform, mental health professionals and individuals.	Mental health issues for which CBT has been proven effective: anxiety, depression, stress and phobias.	
Evaluating the Therapeutic Alliance with a Free‐Text CBT Conversational Agent (Wysa): A Mixed‐Methods Study	Beatty, C., Malik, T., Meheli, S. and Sinha, C.	2022	Frontiers in Digital Health	Mixed‐methods investigation of the therapeutic alliance between a free‐text, CBT‐based conversational agent (Wysa) and users.			AI‐based emotionally intelligent mobile conversational agent app aimed at promoting wellbeing, positive self‐expression and mental resilience using a text based conversational interface.	Anxiety and Depression. 1205 users screen positively on the PHQ‐4 for anxiety or depression symptoms.	
Mental healthcare chatbot based on natural language processing and deep learning approaches: Ted the therapist	Pandey, S., Sharma, S. and Wazir, S.	2022	International Journal of Information Technology	Chatbot called ‘Ted’, developed to help the patients who require support through natural language processing and deep learning approaches.			Ted identifies the intents and contexts from natural language, allowing to interact with the users through appropriate responses for achieve their goals.	Mixed, including suicidal ideation, anxiety and depression.	
A Mental Health Chatbot with Cognitive Skills for Personalized Behavioural Activation and Remote Health Monitoring	Rathnayaka, P., Mills, N., Burnett, D., De Silva, D., Alahakoon, D. and Grey, R.	2022	Sensors	Intelligent chatbot setting using AI to provide recurrent emotional support, personalized assistance and remote mental health monitoring capabilities.		Companion that provides conversational emotional support and continuous personalized engagement (while not attempting to replace existing healthcare services). It is a technological automation that simplifies the BA tasks into an efficient and scalable process.		Anxiety and Depression	
A Virtual Agent to Support Individuals Living With Physical and Mental Comorbidities: Co‐Design and Acceptability Testing	Easton, K., Potter, S., Bec, R., Bennion, M., Christensen, H., Grindell, C. … and Hawley, M. S.	2019	Journal of medical Internet research	Avachat, an autonomous agent for supporting people with comorbid physical LTCs and mental health problems, providing acceptable support and guidance based on self‐management principles.		System structured around a persona (Ava), acting as a focus for the user's interactions through natural language, with which he/she would form something akin to a therapeutic relationship.			20 participants (adults) with Chronic Obstructive Pulmonary Disease (COPD): higher prevalence of condition‐related anxiety and depression and up to 10 times more likely to experience panic attacks than the general population.
Leveraging Natural Language Processing to Study Emotional Coherence in Psychotherapy	Atzil‐Slonim, D., Eliassaf, A., Warikoo, N., Paz, A., Haimovitz, S., Mayer, T. and Gurevych, I.	2024	Psychotherapy		Automatically label clients' utterance‐level emotions during psychotherapy conversations by using state‐of‐the‐art language models for emotion recognition.	Automatic emotion recognition models can be integrated into existing feedback systems to provide an indication of emotional coherence in psychotherapy sessions and allow therapists to modify their interventions accordingly.		872 transcribed sessions from 68 clients in individual psychotherapy. Mixed diagnosis: comorbid anxiety and affective disorders (25.7%), followed by other comorbid disorders (17.1%), anxiety disorders (14.3%) and affective disorders (5.7%). 22.9% of clients had one diagnosis, 20.0% had two and 25.7% had three or more.	
Machine‐Learning‐Based Prediction of Client Distress From Session Recordings	Kuo, P. B., Tanana, M. J., Goldberg, S. B., Caperton, D. D., Narayanan, S., Atkins, D. C. and Imel, Z. E.	2024	Clinical Psychological Science		Developed and evaluated NLP models that automatically predict client symptom ratings (improvement) of a given session based on transcripts of their previous therapy session.	Quickly predict client outcome trajectories and provide therapists with information to tailor and improve quality of care. Highlight how session recordings contain meaningful linguistic signals that could provide contextual information for client and therapist session process ratings.		Mixed; first ten disorders (in order of frequency): anxiety (69.7%), depression (63.3%), academic performance (43.1%), self‐esteem (41.0%), loneliness (36.4%), social anxiety (33.2%), relationship concerns with partner (26.9%), family of origin (21.8%), relationship concerns with friends (19.9%) and body image (17.1%).	
Integrating Bert With CNN and BiLSTM for Explainable Detection of Depression in Social Media Contents	Xin, C. and Zakaria, L. Q.	2024	IEEE Access		To develop three BERT‐based models for depression detection (fine‐tuned BERT, BERT‐BiLSTM and BERTCNN), improving the explainability of the depression detection model.	Assisting mental health professionals in early identification (and intervention) of depression using social media data. Demonstrating the effectiveness of BERT‐based models for depression detection across diverse datasets and conducting a comparative analysis with Mental‐BERT.		Depression	
Automated evaluation of psychotherapy skills using speech and language technologies	Flemotomos, N., Martinez, V. R., Chen, Z., Singla, K., Ardulov, V., Peri, R. … and Narayanan, S.	2022	Behaviour Research Methods		Demonstrate and analyse a platform to process the raw recording of a psychotherapy session and provideperformance‐based feedback according to therapeutic skills and behaviours expressed both at the utterance and at the session level.	The NLP algorithms predicts the behavioural codes reflecting target constructs related to therapist behaviours and skills. The analysis is summarized into a comprehensive feedback report to review the raw MISC predictions of the system, theory‐driven functionals of those, session statistics. The platform can be used as a self‐assessment method as a supportive tool to deliver more effective training.		Not specified (alcohol and poly‐drug abuse)	
Predicting the language of depression from multivariate twitter data using a feature‐rich hybrid deep learning model	Kour, H. and Gupta, M. K.	2022	Concurrency and Computation: Practice and Experience		Designing a hybrid Deep Learning model for predicting the sentiments of depressed online users.		Differentiate depressed and nondepressed users by analysing through sentiment analysis their texts posted on Twitter. The model handles continuous and categorical features and is used as a binary text classification procedure to predict whether or not the text is depressed.	Depression (+ suicide)	
Using Topic Models to Identify Clients' Functioning Levels and Alliance Ruptures in Psychotherapy	Atzil‐Slonim, D., Juravski, D., Bar‐Kalifa, E., Gilboa‐Schechtman, E., Tuval‐Mashiach, R., Shapira, N. and Goldberg, Y.	2021	Psychotherapy		Assess whether topic models could identify clients' functioning levels and alliance ruptures, whether and to what extent the topics identified would change over the course of treatment and whether this change was associated with treatment outcome.	Topic models may enable therapists to be better attuned to specific topics that may signal important events in therapy. The information provided by its outputs allows for conceptual exploration of the therapy process, accessing a summary of topics discussed in a session, locate specific themes associated with rupture or clients' deterioration and direct interventions to improve the situation.		873 sessions from 58 clients in individual psychotherapy. Mixed diagnosis: comorbid anxiety and affective disorders (25.7%), other comorbid disorders (17.1%), anxiety disorders (14.3%) and affective disorders (5.7%). 22.9% of clients had one diagnosis, 20.0% had two and 25.7% had three or more.	
Just in time crisis response: suicide alert system for telemedicine psychotherapy settings	Bantilan, N., Malgaroli, M., Ray, B. and Hull, T. D.	2021	Psychotherapy research		Designed and validated a NLP machine learning model on psychotherapy transcripts to automatically identify and label the level of suicide risk and content expressed by the patient (i.e., risk factors, ideation, method and plan).	Supporting clinical decision and enhancing the response‐time by creating a classification system to assist therapists in suicidality evaluation.		Already available corpus of patient‐therapist transcripts. Data from text communications of 1864 psychotherapy dyads from the telemedicine platform Talkspace	
Natural language processing of clinical mental health notes may add predictive value to existing suicide risk models	Levis, M., Westgate, C. L., Gui, J., Watts, B. V. and Shiner, B.	2021	Psychological medicine		REACH VET (Recovery Engagement and Coordination for Health—Veterans Enhanced Treatment), a machine‐learning‐based suicide prediction model.Evaluated whether REACH VET's ability to predict death by suicide could be improved by including NLP‐derived variables from unstructured EMR data.	Building on established REACH‐VET predictor variables to determine whether linguistic analysis of free‐text clinical notes could improve prediction of death by suicide.		PTSD. The study utilized a cohort of veterans diagnosed with PTSD cohort (because of associations linking PTSD and suicide).	

Appendix S1 explains the categorization used to classify systems.

Appendix S2 reports a summary table of the included studies, showing for each paper its aim(s), sample, intervention/tool, disorder, study design, key results and effect sizes (if present).

### 3.2 Characteristics, Features and Tasks of AI Systems in Clinical Psychology

From the studies reviewed, it can be noted that a range of diverse sets of tools aimed at supporting mental health have been developed. These include intelligent chatbots designed for emotional support and therapeutic guidance, systems for real‐time risk assessment and mood tracking and analytical platforms for assisting clinicians in evaluating therapeutic processes. Together, these systems reflect the growing integration of AI across both user‐facing mental health interventions and backend clinical workflows.

Starting with systems developed as conversational agents, the Behavioural Activation‐based AI chatbot by Rathnayaka et al. (2022) provides personalized conversation, emotional support and remote mental health monitoring. The conversation engine has BA‐oriented NLP modules, divided in (a) feature extractor, for preprocessing and representation learning of the user‐generated text; (b) intent and entity extraction, used to identify and annotate the intention of user utterances; (c) response selection. The emotional support recommends activities (from a bank of common ones and based on the user feedback and mood) that positively impact the user's mood. The remote mental health monitoring is based on Ecological Momentary Assessment (EMA): starting from the emotions expressed in the previous components, it calculates a mood score between 0 and 10 during a seven‐days rolling period by using pre‐trained NLP emotion recognition and sentiment analysis models. This focus on personalized, responsive interactions is also central in other chatbot‐based solutions. Omarov et al. (2023) built an AI‐enabled mobile chatbot psychologist leveraging Artificial Intelligence Markup Language (AIML) and Cognitive Behavioural Therapy (CBT) to offer personalized psychological interventions, while Pandey et al. (2022) built ‘Ted’, a web‐based chatbot using NLP and Deep Learning approaches to help people with mental health‐related issues. In the first one, the use of AIML allows the chatbot to engage users in natural and effective conversations, fostering a sense of connection by understanding their inputs and generating contextually appropriate responses. The chatbot is designed to adapt to the user's needs, detecting their emotional state and providing personalized support accordingly in order to ensure its intervention effectiveness. On the other hand, Ted allows its users to interact through natural language: user messages are lemmatized and pre‐processed before being passed to the Deep Learning model that generates the appropriate response (which also continuously improves over time). Specifically, it identifies the intents and contexts from users' text to interact with them.

Some studies provide data of chatbots with therapeutic aims tested in real‐world interventions. Chiauzzi et al. (2024) adopted Woebot for Mood and Anxiety (W‐MA‐02), a relational agent that guides users through psychotherapeutic content (based on CBT, Interpersonal Psychotherapy [IPT] and Dialectical Behaviour Therapy [DBT]) using text‐based messages. It uses proprietary NLP to help users develop emotion regulation skills in relation to everyday life problems. In particular, it helps users address mood monitoring and management, also employing progress reflection, gratitude journaling and mindfulness practice. Despite not being developed as disorder‐specific, in Chiauzzi et al.'s study it was used to assess depressive or anxiety symptoms changes after 8 weeks of usage participants self‐reporting clinical levels of such symptoms. Similarly, Beatty et al. (2022) investigated Wysa, a free‐text AI‐based mobile conversation agent aimed at promoting wellbeing, positive self‐expression and mental resilience. In particular, they studied the therapeutic alliance between the conversational agent and users, considering that it was demonstrated that a higher engagement leads to improvement in self‐reported depressive symptoms. Using CBT‐based techniques, Wysa provides a therapeutic virtual space where users can discuss their emotions and events in their lives: the conversational agent provides them with AI‐guided listening and empathetic support as well as CBT‐based tools and techniques (such as positive reflection and cognitive reframing) specific to the user's concerns. Wysa is trained in‐house by clinicians and uses interventive conversations created by an internal team. It is able to understand a wide range of emotions, such as uncertainty, disagreement and confusion, from users' text.

Some of the systems developed are tailored to specific populations or comorbidities. Easton et al. (2019) presented Avachat, an autonomous virtual agent system for supporting people with comorbid Chronic Obstructive Pulmonary Disease and mental health problems. The system is structured around a character, called Ava that personifies the support mechanism through a visible and audible presence: it acts akin to a therapeutic relationship for the user to interact with. Similarly, Boggiss et al. (2023) developed COMPASS, a chatbot app intervention for adolescents with Type 1 Diabetes aimed at promoting self‐compassion coping skills through conversational lessons (14 during 2 weeks). The chatbot provides prewritten conversational lessons—based on a standardized 8‐week self‐compassion program and a 2‐week adaptation developed by the authors—using a decision tree ‘rule‐based’ programming. Through ‘quick’ text options it directs the user‐chosen path and using AI it identifies emotions, risk words and the degree of Type 1 Diabetes' management expressed: thus, it is able to deliver tailored and empathetic replies. Furthermore, Berrezueta‐Guzman et al. (2024) explored the integration of ChatGPT into a robotic assistant accompanying children with ADHD in completing their school homework. It has been tested by a panel of therapists working with children diagnosed with ADHD in realistic simulations where the robot would react to specific inputs—such as behavioural events exhibited by the child or commands given through the interface—evaluating its performance across pivotal categories of therapy sessions. Therapists assessed each category by interacting with text‐based questions and simulated events through a dedicated interface offering a realistic simulation.

Expanding beyond chatbots, other systems incorporate broader behavioural modelling and mental state prediction. Kolenik et al. (2024) introduced a computational psychotherapy system for mental health prediction and efficient behaviour change, simulating the theory of mind. Specifically, it targets the non‐clinical population with Stress‐Anxiety‐Depression (SAD) symptoms that have barriers to entry into the mental healthcare system. The system uses AI and ML (but not only) to understand its users by building various idiographic, detection and forecasting models combined with novel ontologies on mental health and behaviour change. Through LLMs, the system generates linguistic outputs as a response to the input text in the form of motivational messages personalized to the user's personality. Moreover, at the end of a conversation, the system re‐evaluates the user's well‐being post‐support to provide further tailored strategies.

Differently, other AI systems serve more as clinical tools to support professionals. An example comes from Flemotomos et al. (2022), who developed a platform for processing the raw recording of psychotherapy sessions and providing timely performance‐based feedback aligned to therapeutic skills and behaviours expressed both at the utterance and at the session level—which reflect target constructs related to therapist behaviours and skills. Such platform can be used by the therapist as a self‐assessment method or by a supervisor as a training supportive tool. In detail, NLP algorithms predict behavioural codes from the linguistic information of sessions' transcription. The behavioural analysis is then summarized into a comprehensive report, delivered on an interactive web platform through which the therapist can: review the raw Motivational Interviewing Skill Code (MISC) predictions of the system (e.g., empathy score and utterances labelled as reflections) and the related theory‐driven functionals (e.g., ratio of questions to reflections); session statistics (e.g., ratio of client's to therapist's talking time); take notes and make comments linked to specific timestamps or utterances.

Remaining in the context of therapy evaluation and monitoring, Atzil‐Slonim et al. (2021) assessed the potential of topic models to identify clients' functioning levels and alliance ruptures by examining therapy sessions transcripts: in particular, if and to what extent the topics changed over time and if this change was associated with treatment outcome. It functions by adopting latent Dirichlet allocation that, employing Bayesian probabilistic modelling, finds clusters of terms (topics) that tend to co‐occur in subsets of the transcripts. Similarly, Kuo et al. (2024) developed and evaluated NLP models for predicting client symptom ratings of a therapy session, based on transcripts of their previous one. In particular, they employed RoBERTa as the primary representation of the texts, which can take sentences or paragraphs and output numeric vectors that can be used for prediction tasks. Following on predictive tasks, Levis et al. (2021) employed REACH‐VET to evaluate its capability in predicting death by suicide in veterans with a PTSD (Post Traumatic Stress Disorder) diagnosis by integrating NLP‐derived variables in analysing free‐text clinical notes. These latter were processed by Sentiment Analysis and Cognition Engine [SÉANCE], a Python‐based NLP package. Further exploring suicide prediction in clinical populations, Bantilan et al. (2021) designed and validated an NLP model to automatically detect, from psychotherapy transcripts, the level of suicide risk expressed by the patient and related contents (i.e., risk factors, ideation, method and plan). Trained on large‐scale clinical data from a tele‐behavioural health provider, the most accurate model scored patient texts every 30 min, updating the suicide risk score linked with a therapist‐client transcript as the psychotherapy proceeded.

Again, from therapy transcripts and conversions other studies focus on emotion recognition and mental state classification. Atzil‐Slonim et al. (2024) employed state‐of‐the‐art BERT‐based language models (and their corresponding lightweight adapter solutions) to automatically label clients' utterance‐level emotions during psychotherapy conversations. In detail, they fine‐tuned several BERT‐based models for patients' emotion recognition through text. Extending this direction, Xin and Zakaria (2024) developed three BERT‐based models for depression detection and compared them with MentalBERT, in order to evaluate their effectiveness against a state‐of‐the‐art benchmark tailored for mental health applications. Aiming to explain how the models work and perform their decision‐making process, the authors employed a comprehensive and intuitive interface. After the user submits the text, the depression detection model processes the input and decides if it is a signal of depression. It does so by using colours and related intensity to display the importance for each word in the input text. Lasty, Kour and Gupta (2022) designed a hybrid Convolutional Neural Network and Long‐Short Term Memory (CNN‐LSTM) Deep Learning model useful for predicting sentiments related to depression—this time in online users—distinguishing depressed and non‐depressed ones. The online users' text posted on Twitter are examined through sentiment analysis. This hybrid system can extract deep features from sentences, based on their semantic and syntactic properties: LSTM is able to manage both “vanishing gradient” and “exploding gradient” problems and can learn the word‐level semantic information.

### 3.3 Beneficial Effects of AI Systems for Patients' Mental Health and Practitioners Work in Clinical Psychology

The selected studies highlight the potential benefits that the use of AI could offer to both users' mental health and practitioners' work.

Starting with user‐facing systems that prioritize direct psychological support and emotional engagement, the Behavioural Activation‐based chatbot by Rathnayaka et al. (2022) was effective in supporting users with mental health issues, in particular by providing personalized interactions, scheduling actions and tracking mood changes. It was able to enhance mood awareness and self‐reflection, as reported by one of the users: ‘By using the app, I am more aware of how my moods fluctuate. [...] alleviated some negativity I was experiencing at the time’. Similarly, the study by Beatty et al. (2022) showed that Wysa users were satisfied with the use of the tool, as it provided a safe, comfortable and supportive environment—elements that are all considered necessary for the development of the therapeutic alliance. In the study by Berrezueta‐Guzman et al. (2024), the ChatGPT‐based support tested seemed to be effective in enhancing therapy: in particular, it adapted to each child's needs and therapeutic progress by offering tailored interventions and interactions. With interactive dialogue and gamified sessions, it helped keep children engaged and motivated, thereby increasing the overall effectiveness of the therapy. The COMPASS system by Boggiss et al. (2023) demonstrated notable benefits for users by offering personalization, self‐management support, ease of use and connectivity with others, making it a valuable tool for enhancing well‐being. Furthermore, Ted, developed by Pandey et al. (2022), offered significant benefits for users, helping them reduce the stigmatization often experienced when interacting with professionals: in particular, it enables users to interact naturally, generating appropriate responses based on their input, thus offering a more comfortable and confidential means of seeking help. Indeed, it offers a promising alternative to traditional therapy for patients, providing additional support and resources to those in need and enabling them to take charge of their mental well‐being in an accessible, cost‐effective and efficient way. Similarly, the system by Easton et al. (2019) enhances users' self‐management skills, offering immediate support and in turn increasing accessibility and availability. In particular, patients positively value the peer‐driven support as well as the emotional well‐being advice, the behaviour change techniques provided and the triangulation of clinically accurate information. Participants reported that the system was actually supportive, providing personalized interventions adapted to the specific user's emotional and cognitive profile. Indeed, it was able to provide more effective stress and anxiety relief compared to state‐of‐the‐art alternatives as well as the same capacity to alleviate depression symptoms. In a similar manner, the system by Chiauzzi et al. (2024) showed a significant reduction both in self‐reported depressive symptoms and anxiety symptoms of patients across the intervention period (starting from an elevated level of them). In the study by Xin and Zakaria (2024), where BERT was integrated with Convolutional Neural Network (CNN) and Bidirectional Long‐Short Term Memory (BiLSTM) for the detection of depression by analysing social media content, these models were highly effective in their task, thus enhancing the reliability of depression diagnoses. For users, these models enable timely interventions and support for individuals suffering from depression. Lastly, the NLP model implemented by Bantilan et al. (2021) provides an accurate assessment of individual suicide risk at the sentence level, an individualized approach that can help ensure that therapy is tailored to the unique needs of each patient, promoting more personalized and potentially life‐saving interventions.

Moving to the effects for practitioners (professionals, psychologists and therapists working in clinical psychology), the remote‐control mood tracking described by Rathnayaka et al. (2022) was useful in the changing behaviour process by detecting dangerous conversations. For professionals, ChatGPT generates insights into therapeutic outcomes, offering guidance that can inform and improve future treatment strategies (Berrezueta‐Guzman et al. 2024). COMPASS (Boggiss et al. 2023) provided clinical utility by complementing standard care, particularly during the pandemic. Ted (Pandey et al. 2022) reduces the resources and time required for training, ultimately decreasing the workload for them. The AI‐enabled mobile chatbot psychologist developed by Omarov et al. (2023) moves in the same direction: by providing personalized psychological support, it helps to reduce the burden on mental health professionals, serving as a valuable adjunct to existing mental health services. The system by Chiauzzi et al. (2024) can be integrated with concurrent mental health treatments as well as detect crises with resource delivery. Benefits for professionals also come from the system by Flemotomos et al. (2022), which could provide fast and low‐cost feedback to them, in turn improving the quality of services and more positive clinical outcomes. Indeed, performance‐based feedback is essential for practitioners, both for training new ones and for maintaining the already acquired skills. It can also be used for evaluation, keeping in mind the degree of reliability of the system itself. Additionally, practitioners could use the system by Kolenik et al. (2024) to monitor their users, considering the reliability of its diagnosis, thus reducing their workload burden. Moreover, their system featured a forecasting ability useful to predict the likelihood of future depressive episodes (up to 7 days in advance) and intervene in a timely manner. In line with the latter, the study by Levis et al. (2021) suggests that the NLP‐derived variables added in REACH VET could help in better identifying and monitoring suicide risk over time, leading to more timely and targeted interventions. For professionals, this enhanced predictive capability offers more precise insights into patients' distinct risk sensitivities, ultimately supporting more effective treatment strategies. The system by Bantilan et al. (2021) helped professionals in identifying critical situations, alerting telehealth clinicians to potential suicide risk in a patient's content and allowing them to provide timely crisis resources. In the study by Kour and Gupta (2022), the use of this feature‐rich hybrid deep learning model can enhance the diagnostic process by providing deeper insights into the behavioural and clinical aspects of depression, ultimately supporting more effective treatment planning and decision‐making. The system by Atzil‐Slonim et al. (2024) offers an opportunity for professionals to examine emotional processes on a larger scale and with higher specificity, improving their ability to understand and intervene in patients' emotional experiences. Practitioners can benefit from being more receptive to subtle expressions of positive emotions and can tailor their interventions to help clients better align their emotional experiences with their verbal expressions. Similarly, in the study by Easton et al. (2019), the empathetic ability of the system to identify and react to non‐verbal clues from people's text will be pivotal to enhance the therapeutic relationship between agent and patient. The ML model by Kuo et al. (2024) improves therapist feedback and helps predict treatment outcomes. The development of NLP models to predict client symptoms from session recordings shows promising results, with potential for integration into outcome‐monitoring systems, ultimately enhancing the quality of care provided. Lastly, tool by Atzil‐Slonim et al. (2021) is confirmed to be effective in supporting therapists by providing a summary of topics discussed in a session, enabling them to identify themes related to alliance ruptures or clients' deterioration. This helps them to orient their interventions more effectively, in turn improving clients' functioning. Additionally, the thematic model can be integrated with existing monitoring tools, allowing therapists to track significant language processes during therapy sessions.

## 4 Discussion

This study aimed to systematically review the existing literature regarding the use of AI in clinical psychology to improve psychological interventions for DMH. AI systems and solutions have been increasingly integrated into DMH care to enhance the processes of prevention, diagnosis, intervention and monitoring of digital mental health as well as to personalize interventions and provide immediate support for individuals with psychological distress. The potential of AI lies in its ability to process large amounts of data, detecting patterns in patient behaviours and symptoms and providing interventions tailored to users' psychological needs. Due to this, AI systems have proven effective in providing clinicians with deeper insights into patient symptomatology and complementing traditional clinical assessments and interventions.

A systematic review of studies (dated from 2019 to 2024) related to this topic was conducted, focusing on the task performed by AI systems, specifically with NLP and Machine Learning (ML) features. The selected studies were related to research about clinical conversations between patients and their therapists or texts generated in clinical settings, also showing the benefits for patients' mental health and practitioners' work. In the previous section, the results of this review have been described to now provide some related considerations.

AI systems in psychological interventions include DMH apps, therapeutic chatbots and wearable monitoring devices. Particularly relevant AI applications are therapeutic chatbots, which leverage NLP to process users' linguistic and emotional input to provide them with structured psychological support in response to the detected mental states. Systems such as Wysa adopt Cognitive‐Behavioural Therapy (CBT) techniques to assist users in managing their emotions and improving psychological resilience through personalized interventions. Wysa, in particular, has demonstrated efficacy in enhancing therapeutic alliance and improving self‐reported depressive symptoms, especially when engagement levels are high (Beatty et al. 2022). Similarly, chatbots like the Behavioural Activation‐based system by Rathnayaka et al. (2022) use NLP‐driven modules to personalize conversations and monitor users' moods over time using EMA. These systems integrate sentiment and emotion recognition to generate dynamic mood scores and suggest mood‐improving activities, reinforcing emotional self‐awareness and adaptive behaviour. AI systems diagnostic tools have also emerged as a valuable resource for the diagnostic process. They facilitate the detection and classification of psychological disorders through ML algorithms capable of analysing linguistic patterns and behavioural indicators. For instance, systems such as those developed by Xin and Zakaria (2024) and Kour and Gupta (2022) utilize BERT‐based or hybrid deep learning models to perform sentiment analysis on user‐generated text—such as social media data—to detect depressive symptoms with high accuracy. Similarly, AI models that incorporate text‐based sentiment analysis have been used to predict depressive symptoms, enhancing early detection and intervention strategies. These predictive models, including those employed by Bantilan et al. (2021) and Levis et al. (2021), also enable risk assessment for suicide based on clinical notes or therapy session transcripts, thus supporting both crisis detection and personalized care delivery.

Considering their characteristics and applications, from the studies reviewed it emerges that the implementation of AI systems could have both benefits and challenges. First and foremost, these digital technologies could help to overcome financial and geographical barriers, thus improving access to psychological support to a wider range of people (Gual‐Montolio et al. 2022; Naslund et al. 2020). Chiauzzi et al. (2024) have shown that DMHIs provided by using phones can be effective for reducing anxiety and depression levels. Young adults and adolescents are the ones showing the higher acceptance of DMH (Rideout et al. 2018): in fact, by allowing individuals to seek help without direct exposure, they help to reduce the stigma often associated with DMH issues (Grist et al. 2019). Lastly, DMH enables continuous and real‐time monitoring that allows for providing prompt interventions.

However, the implementation of AI in clinical psychology has not only advantages. The effectiveness of DMH tools varies across populations and while some studies have demonstrated significant improvements in psychological well‐being, others have reported less conclusive findings. For example, Ogawa et al. (2022) and Kolenik and Gams (2021) found no statistically significant changes in symptom reduction following chatbot interactions. These preliminary findings suggests that further refinements are needed to optimize these technologies. Furthermore, one of the main challenges and issues is the lack of standardization and scientific validation: indeed, Leigh and Flatt (2015) found that less than 5% of DMH apps had been adequately validated. Moreover, some studies do not compare their results with control trials or groups (Chiauzzi et al. 2024; Gual‐Montolio et al. 2022)—an already rarely performed process in psychotherapy—thus reducing their robustness and making the application of such AI systems only self‐referential. In turn, these issues could diminish overall reliability and replicability of the study (Gual‐Montolio et al. 2022). Indeed, the absence of control groups makes it difficult to determine whether observed improvements are due to the AI intervention itself or, instead, to external, nonspecific factors such as expectancy effects, time or user motivation. This limitation could significantly weaken the robustness of the (still preliminary) evidence base and the validity of clinical outcomes (Andersson et al. 2019). In addition, AI systems could produce biases due to their training that could lead to mistakes in diagnosis or ineffective interventions for some individuals. For example, findings from some studies show that AI could indeed detect early signs of mental disorders or psychological difficulties through social media analysis (Guntuku et al. 2019; Taccini and Mannarini 2024), but others show how AI systems can produce false positives (Haghish and Czajkowski 2024) and fail to recognize symptoms in some populations (Rai et al. 2024). A prime example comes from Mehrabi et al. (2021), showing that AI diagnostic tools focused on depression may be less accurate in relation to ethnic minorities. This is a very important issue for health assessment (also gender, age, etc.). In fact, the literature in general (Moudden et al. 2025; Muntaner et al. 2013; Sellers et al. 2009; Snowden 2003; Williams 2003) has explored this issue in depth. With regard to the contribution proposed here, it should be noted that the selected articles do not explore this issue, which should certainly be considered in future studies. Such variability in outcomes underscores the need for further research to ensure their effectiveness across diverse user groups. Indeed, the stronger effectiveness reported for anxiety and depression‐related interventions may be linked to the extensive scientific knowledge of such disorders: over the years, these conditions have been deeply studied and conceptualized within structured diagnostic and therapeutic frameworks, making them more amenable to algorithmic modelling and in turn facilitating the creation of effective AI systems (Kazdin and Rabbitt 2013). In contrast, more complex or less codified psychological issues—such as personality disorders, relational trauma or comorbid presentations—pose a greater challenge for algorithmic tractability (Topaz and Pruinelli 2017). Therefore, current findings may reflect more the maturity of scientific understanding in these specific domains than the general applicability of AI across all mental health conditions. This last observation also highlights that AI systems are usually—if not always—bound to deal with a single specific disorder in a predefined way, based on the data and techniques they are trained on. Referring to the studies reviewed, the psychological disorders mainly addressed were anxiety and depression, followed at a distance by suicidal ideation, PTSD, stress and affective disorders. In relation to the psychological techniques implemented, most followed behaviour‐related approaches, such as CBT (predominantly), DBT or Behavioural Activation (BA), and only a small portion referred to other like theory of mind, schema therapy or short term psychodynamic psychotherapy. Thus, even though their responses‐suggestions may be tailored to the users' peculiar situation, they cannot aid if other symptoms or criticalities arise. This could affect not only clinical trials, which suffer in terms of data shareability and replicability of results (Smith et al. 2023), but also clinical intervention, inasmuch as some situations may require multiple of them. Thus, hybrid models where the therapist's supervision is assured and AI acts as support (rather than a replacement) may be ideal Topol (2019). Indeed, as emphasized by Flemotomos et al. (2022), AI‐based technologies are best positioned as assistive, augmenting the capabilities of clinicians rather than replacing them. Misinterpretation of automatically generated feedback or uncritical acceptance of AI‐driven conclusions could have serious implications for patient care. It is therefore vital that users are trained to understand the scope and limits of these tools and that human oversight remains central to any clinical deployment. Conversely, an excessive dependence and reliance on AI risks reducing human supervision. If this latter is overlooked, automated clinical praxis may lead to misdiagnosis or rigid intervention plans, potentially compromising mental health care quality (Floridi et al. 2018; Torous et al. 2019).

Last but not least, AI systems are less understanding of human feelings and struggle to develop empathetic relationships with patients (Bickmore and Picard 2005): while some chatbots like Woebot and Wysa provide effective CBT interventions (Fitzpatrick et al. 2017), they are not able to fully replicate human emotional understanding and connection (Bickmore and Picard 2005), which is a key element in therapy that provides long‐term therapy success (Naslund et al. 2020). So, this leads to a higher and quicker abandonment of digital programs compared to therapy provided by human professionals (Baumel et al. 2019). This latter also guarantees a correct data treatment, which is crucial for building a relationship of trust from the patient, while using AI systems leaves some ethical concerns (Smith et al. 2023). Indeed, AI‐driven interventions collect and process sensitive people's DMH data that need secure storage and protection from unauthorized access. Regulations like GDPR and HIPAA exist, but a unified ethical framework for AI in psychology is missing and gaps related to its implementation remain (Ruggieri et al. 2021). For example, the EU has a more careful approach compared to the United States and China, which are more permissive: this leads to debates over appropriate governance (Jobin et al. 2019). Table 3 reports a brief comparison between regional regulatory approaches with some related practical recommendation for clinicians.

**TABLE 3:** Regional regulations and related practical recommendations for clinicians.

Region (framework)	Key regulatory principles	Practical recommendations for clinicians	Consent wording recommendations	Data storage and security checklist
EU (GDPR)	‐ Lawful basis for processing (Art. 6); special category data: health and genetic (Art. 9); right to access, rectification and erasure; data minimization and purpose limitation	Clinicians must ensure patients understand data use: Explicit, informed consent required for sensitive health data and must include withdrawal options. Use secure electronic health record systems compliant with GDPR.	*I consent to the collection, storage and use of my health data for the purpose of my clinical care and related research*.	‐ Data pseudonymization/anonymization; encryption in transit and at rest; access controls (role‐based); audit logging.
USA (HIPAA)	‐ Protected health information (PHI); privacy rule: patient rights to access and correct PHI; security rule: administrative, physical and technical safeguards	Clinicians must provide notice of privacy practices: Consent for use and disclosure of PHI (for treatment, payment and operations). Train staff on HIPAA compliance.	*I authorize the use and disclosure of my health information as described in this notice for treatment, payment and healthcare operations*.	‐ Encrypt PHI in storage and transmission; limit access to minimum necessary; regular risk assessments; secure backup and disaster recovery.
UK (NHS and Data Protection Act 2018)	‐ Aligns with GDPR; confidentiality and Caldicott principles; explicit consent for processing sensitive data	Follow Caldicott Guardian recommendations and document consent in patient record: Use plain language consent forms; Explain data sharing with secondary uses (research and audits).	/	‐ Secure NHS systems; role‐based access; data sharing agreements for research.
Canada (PIPEDA and Provincial Laws)	‐ Personal Information Protection and Electronic Documents Act—Health information acts vary by province	Clinicians must follow provincial health information regulations: Must obtain meaningful consent. Ensure cross‐border data transfers comply with law.	*I consent to the collection, use and disclosure of my health information for my care and as required by law*.	‐ Access restrictions; ‐ Audit trails; ‐ Secure electronic systems.
Australia (Privacy Act 1988 and My Health Records Act)	‐ Australian Privacy Principles (APPs); special rules for health information—Patient right to access and correct	Clinicians should check patient consent before sharing info and keep accurate audit records:‐ Explicit consent for My Health Record uploads.	*I consent to my health information being included in My Health Record and shared with healthcare providers involved in my care*.	‐ Secure My Health Record systems;‐ strong authentication;‐ staff training on privacy.
Other frameworks (WHO guidance, ISO 27799)	‐ WHO: Health data should be confidential, secure and used for care/research ethically; ISO 27799: Guidelines for information security management in health	Useful for institutions without strong national laws. Integrated with EHR policies. Use clear, informed consent; document consent digitally or on paper.	/	‐ Risk assessment; encryption and access control; incident response plan.

Thus, issues of data ownership and patient consent are concerning (Huckvale et al. 2019), particularly in AI systems that continuously monitor users (Murdoch 2021). Also, many patients are not aware of how AI algorithms function and collect data; therefore, an ethical AI use demands clear, informed consent and potentially dynamic consent models (Luxton 2014; Sharkey and Sharkey 2021). This scenario highlights the need to develop clear and recognized (by the scientific community) protocols for the use of therapeutic chatbots and AI‐driven interventions within psychological interventions, designed to protect human health while ensuring the responsible deployment of AI.

This is in line with several recommendation provided by the American Psychological Association (APA): indeed, they suggest to: (1) not rely on generative AI to deliver psychotherapy and psychological treatments; (2) protect users from misinformation, algorithmic bias and illusory effectiveness; (3) create specific safeguards for vulnerable populations and (4) implement comprehensive AI and digital literacy education (APA 2025). Regarding point 1, APA states that GenAI chatbots and app should not be used in substitution to a qualified therapist but only as a support, inasmuch as relying solely on them may pose several risks, such as risk of bias and misinformation, misrepresentation of services, creating a false sense of therapeutic alliance and incomplete assessment. Thus, they recommend specific training on these emerging technologies as well as following the available ethical guidelines (although still not fully adequate to the reality of using AI for promoting mental health) and asking users about their use of such apps. In line with this, regarding Point 2 the APA also suggest to educate patients on algorithmic bias, since this digital tools (and especially general‐purpose models) are trained to agree to users. In particular, clinicians should pay particular attention to the use of GenAI chatbots and apps among vulnerable populations (Point 3—e.g. adolescents, socially isolated individuals and people with confirmed diagnosis), because these could act as powerful amplifiers of already existing vulnerabilities or issues. Thus, therapist and mental health expert should learn and know how such AI‐based tools work, in order to be aware of their potential misuse, reduce the possible negative effects and maximize their benefits for patients (APA 2025).

A key ethical dilemma could arise: in order to safely use AI systems and tools, it is necessary to understand how they work—yet to understand them it may need to expose users to them, thereby potentially putting their well‐being at risk. This epistemological paradox calls for rigorous, ethically sound experimentation frameworks that safeguard participants while advancing scientific knowledge (Mittelstadt 2019; Morley et al. 2020). Indeed, without such standards the clinical application of AI remains vulnerable to misuse or unintended harm.

In conclusion, some questions remain unanswered: can AI adapt to specific individual psychological needs? And can it provide adequate care? Current AI systems work on predefined models, which limit their flexibility. Conversely, Adaptive AI raises concerns about privacy and algorithmic over‐personalization (Mandal et al. 2025). However, it is also true that, in global regions where mental health professionals are lacking, AI therapy might be the best, if not the only option available (van Heerden et al. 2023).

The preliminary findings from the reviewed studies show that AI can most definitely improve DMH access, but it must remain inclusive, transparent and human‐supervised to ensure patient rights and clinical integrity. Ultimately, while AI technologies hold immense promise for enhancing psychological practice, their development and implementation must be governed by a set of rules and professional standards internationally shared by the scientific community. This echoes the historical evolution of psychological assessment tools: decades of scientific effort were dedicated to building standardized protocols for test administration, scoring and interpretation, where the establishment of clear operational criteria and validation methods was essential for the legitimacy and ethical use of those instruments (Hunsley and Allan 2019; Institute of Medicine 2015). A similar path now lies ahead for AI in mental health: shared rules must be developed to define how, when and by whom these tools can be used, with the aim of ensuring both clinical efficacy and the protection of human health (He et al. 2019; Sebastian et al. 2020). In light of this, fostering an ethical AI integration in psychology requires collaboration between psychologists, AI researchers, ethicists and policymakers.

In light of all this, the potential of AI in the clinical setting clearly emerges as well as its current limitations. It is clear that AI use must be governed and not just supervised by individual professionals. This implies the possibility of creating global scientific protocols for AI use in clinical settings, with rules that can guide professionals—but also developers—aimed at sharing clear and agreed criteria for the development and use of such tools. In particular, rules that are not only oriented towards technological development but also towards the ultimate goal of improving and preserving human health are needed.

## 5 Conclusions

The use of AI and NLP definitely holds transformative potential for clinical psychology, offering new avenues for enhancing mental health care delivery. Using AI in clinical psychology can provide more accurate data for anticipating how therapy is going to develop. Moreover, thanks to improved accessibility to them, AI systems for DMH can be used to reach more people than in‐person therapy, using all patients' data to monitor the progress of their symptomatology and adjust the intervention. Through tools such as AI‐enabled chatbots and emotion recognition systems, NLP can support scalable, cost‐effective and immediate psychological interventions. These technologies not only help to reduce the burden on traditional services but also contribute to the destigmatization of mental health care by increasing accessibility and personalization of support. Furthermore, NLP‐based models demonstrate the capability to extract meaningful linguistic signals from therapy sessions, providing clinicians with timely, contextualized insights to tailor interventions and improve the quality of care. The integration of ML into psychotherapy research—via tools like emotion annotation, session trajectory prediction or rupture detection—suggests promising pathways for augmenting traditional therapeutic processes.

Nonetheless, the current state of AI and NLP technologies in clinical psychology is marked by significant limitations. Models are often developed and validated in constrained environments, limiting their generalizability across diverse populations and therapy contexts. NLP tools still lack the capacity to interpret non‐verbal and paraverbal communication cues, which are central to therapeutic interactions. Ethical, technical and cultural gaps remain in the application of AI, particularly in understanding nuanced emotional dynamics and non‐verbal aspects of client expression. These criticalities underscore the importance of continued research and longitudinal evaluation, particularly across varied socio‐demographic groups. Crucially, while NLP systems can offer valuable support, they must not be seen as substitutes for human clinicians. Human supervision is essential—not only in interpreting the output of these tools but also in ensuring that automated systems are used responsibly and ethically. Moreover, all the sensitive and personal information provided by patients in therapy needs strict security measures in order to prevent privacy breaches. Thus, using AI systems in clinical psychology does not come without ethical issues linked to data privacy, transparency as well as to the quality and effectiveness of their interventions. Early‐stage co‐design with clinicians and patients could guide the development of these technologies in socially responsible and clinically meaningful ways. Ensuring that AI systems are interpretable, transparent and integrated into clinical supervision practices will help mitigate risks and foster trust. As the field evolves, a sceptical, critical approach towards machine‐generated outputs will not hinder progress; rather, it will serve as a catalyst for refining these technologies and deepening their integration into practice.

Therefore, it is paramount to avoid a complete delegation of the intervention to AI; conversely, its use should be strictly supervised by professionals and integrated with an ethical approach that ensures patient well‐being and data protection. As AI continues to evolve, its potential to enhance DMH care remains vast. However, ensuring its ethical implementation, optimizing intervention efficacy and addressing existing limitations will be crucial in maximizing its benefits for both practitioners and patients. In conclusion, NLP technologies are poised to significantly enhance psychological care but their successful and ethical implementation demands robust human involvement. Supervision, interpretation and ethical scrutiny by trained professionals are not optional safeguards—they are fundamental requirements for aligning technological innovation with therapeutic integrity and patient well‐being.

## Conflicts of Interest

The authors declare no conflicts of interest.

## Supporting information


**Appendix S1:** Explanation of the categorization used to classify systems.

**Appendix S2:** Summary table with included studies.

## Data Availability

Data sharing not applicable to this article as no datasets were generated or analysed during the current study.
