# Supplementary material for: The Role of Artificial Intelligence in Clinical Psychology: How AI and NLP Systems Are Reshaping Psychological Interventions. A Systematic Review
Source: Clin Psychol Psychother. 2026 Feb 25;33(2):e70242. doi: 10.1002/cpp.70242 (PMC12933282; doi:10.1002/cpp.70242)
Supplement: Supplementary file 1 — Appendix S1: Explanation of the categorization used to classify systems. [file CPP-33-e70242-s001.docx]

**Appendix 1: explanation of the categorization used to classify systems**

1. Main target of the system
   1. Patients: the system is used by patients or its directed toward patients;
   2. Therapist: the system is used by clinicians or its directed toward clinicians.
2. Type of intervention
   1. Supportive: the purpose of the system is to support the intervention or to accompanying during the intervention;
   2. Substitutional: the purpose of the system is to perform a task/offer a service in place of the clinician.
3. Type of disorder
   1. Psychological: explicitly related to mental health conditions only;
   2. Psychological on medical-base: related to mental health conditions deriving from issues on a medical/anatomical/biological/physiological dimension.

*Acronyms list*

CBT: Cognitive-Behavioral Therapy

IPT: Interpersonal Psychotherapy

DBT: Dialectical Behavior Therapy
