# Supplementary material for: The Role of Artificial Intelligence in Clinical Psychology: How AI and NLP Systems Are Reshaping Psychological Interventions. A Systematic Review
Source: Clin Psychol Psychother. 2026 Feb 25;33(2):e70242. doi: 10.1002/cpp.70242 (PMC12933282; doi:10.1002/cpp.70242)
Supplement: Supplementary file 2 — Appendix S2: Summary table with included studies. [file CPP-33-e70242-s002.docx]

**Appendix 2: summary table with included studies.**

| Title | Aim | Sample (n, population) | Intervention/tool | Targeted disorder | Study design | Key outcomes/ measures | Effect size |
| --- | --- | --- | --- | --- | --- | --- | --- |
| Demographic and clinical characteristics associated with anxiety and depressive symptom outcomes in users of a digital mental health intervention incorporating a relational agent | Evaluated Woebot for Mood and Anxiety (W-MA-02). | 256 participants, convenience sample (18+, U.S. residents, English literacy, no current suicidal ideation or previous suicide attempt, no diagnosis od psychotic or bipolar disorder, no previous use of a Woebot application) | Woebot for Mood and Anxiety (W-MA-02), an intervention delivered via Woebot, a relational agent that utilizes thoughtful conversational design and some NLP to incorporate elements of CBT, Interpersonal Psychotherapy (IPT), and DBT into a text-based interface on a smartphone app. | Depressive or anxiety symptom. | Non-randomized, single-armed, and open-labeled exploratory study. | Both the depressive and anxiety subsamples had significant reductions in symptoms at Week 8. | Depressive subsample: Cohen’s d = - 1.23.  Anxiety subsample: Cohen’s d = - 1.24 |
| Computational psychotherapy system for mental health prediction and behavior change with a conversational agent | Presents a computational psychotherapy system for mental health prediction and behavior change using a conversational agent. | 42 participants, convenience sample. | Novel artificial cognitive architecture mimicking theory of mind. Composed of three modules: Natural language processing module, Theory of mind module, Natural language generation module. | Non-clinical population with SAD (stress, anxiety, depression) symptoms. | Computational experiments and empirical interventional study. | The system outperformed state-of-the-art systems in terms of the number of detected categories and detection accuracy. | / |
| Future of ADHD Care: Evaluating the Efficacy of ChatGPT in Therapy Enhancement | Evaluate ChatGPT’s effectiveness in therapy settings for ADHD nonpharmacological therapies to integrate AI into healthcare responsibly. | 10 participants, experts in therapies for children with ADHD. | Custom ChatGPT. | ADHD. | Therapeutic validation, using the Delphi method. | Findings highlight the potential for ChatGPT to significantly improve ADHD care, as well as the need for enhancements in privacy, cultural sensitivity, and interpreting nonverbal cues its effective healthcare integration. | / |
| Improving the Well-being of Adolescents With Type 1 Diabetes During the COVID-19 Pandemic: Qualitative Study Exploring Acceptability and Clinical Usability of a Self-compassion Chatbot | Evaluate the acceptability and potential clinical utility of COMPASS among adolescents aged 12 to 16 years with T1D and diabetes health care professionals. | 30 participants, 19 adolescents with T1D and 11 diabetes health care professionals. | COMPASS chatbot, designed to deliver daily content in 14 conversational lessons daily across 2 weeks, aimed at facilitating self-compassion coping skills for adolescents with T1D. | Psychological impacts in adolescents with T1D. | Qualitative study, using focus groups and one-on-one interviews. | Findings suggest that COMPASS is acceptable, relevant to common difficulties, and has clinical utility. Desired features include more personalization, self-management support and breadth and flexibility of tools. | / |
| Artificial Intelligence Enabled Mobile Chatbot Psychologist using AIML and Cognitive Behavioral Therapy | Presents an AI-enabled mobile chatbot psychologist that leverages Artificial Intelligence Markup Language (AIML) and Cognitive Behavioral Therapy (CBT) to offer personalized psychological interventions. | N.D. | The proposed chatbot psychologist employs AIML to understand user inputs and generate contextually appropriate responses. The chatbot is equipped with a knowledge base comprising CBT principles and techniques, enabling it to provide targeted psychological intervention to address a wide range of mental health issues (e.g. anxiety, depression, stress, and phobias). | Varying levels of psychological distress. | Experimental. | Results demonstrate the chatbot's ability to deliver personalized interventions, with users reporting significant improvements in their mental well-being. The main challenges relate to privacy concerns, ethical consideration, ongoing support and maintenance, and over-reliance. | / |
| Evaluating the Therapeutic Alliance with a Free-Text CBT Conversational Agent (Wysa): A Mixed-Methods Study | Examine whether users perceive a therapeutic alliance with an AI conversational agent (Wysa) and observe changes in the therapeutic alliance over a brief time period. | 1205 participants, eligibility was determined by scoring ≥3 for the first two questions (anxiety) or ≥3 for the second two questions (depression) of the PHQ-4 Questionnaire. | Wysa is an AI-enabled mental health app that leverages evidence based cognitive-behavioral techniques in the user interface and the interventions within the CA. It is designed to provide a therapeutic virtual space for user-led conversations through AI-guided listening and support, access to self-care tools and techniques including CBT-based tools, as well as one-on-one human support. | Anxiety or depression symptoms. | Mixed method approach. | No significant difference in the alliance scores. Users’ conversation revealed elements of bonding such as gratitude, self-disclosed impact, and personification with the CA. Users’ therapeutic alliance scores improved over time. | / |
| Mental healthcare chatbot based on natural language processing and deep learning approaches: Ted the therapist | Propose an AI web based chatbot called ‘‘Ted’’ to assist people with mental health-related queries. | / | TED, a chatbot based on NLP and deep learning: it allows the users to interact, use natural language to take input, and generate the appropriate response according to the input. | / | Comparative analysis. | The accuracy of TED is 98.13% in providing the appropriate response. | / |
| A Mental Health Chatbot with Cognitive Skills for Personalised Behavioural Activation and Remote Health Monitoring | Present the design and development of a BA-based AI chatbot offering continuous personalised engagement and emotional support, and its participatory evaluation in a pilot study setting. | 34 participants, convenience sample. Among the 6 inclusion criteria, there were i) using the app to answer at least two valid PHQ2 surveys; ii) using the app to perform at least two “feelings check-ins”. | BA-Based AI Chatbot (called “Bunji”) implemented as a cross-platform smartphone application and designed as a series of chatbot capabilities grouped into: (1) personalised conversation, (2) emotional support, (3) remote mental health monitoring. | Mental health issues, and in particular anxiety and depression. | Participatory evaluation. | Results confirmed its effectiveness in providing support for individuals with mental health issues. | / |
| A Virtual Agent to Support Individuals Living With Physical and Mental Comorbidities: Co-Design and Acceptability Testing | Explore the acceptability of an autonomous agent for supporting people with comorbid physical LTCs (COPD in particular) and mental health problems. | 12 participants: 5 people with COPD, 1 caregiver, 6 health professionals. | Avachat, a virtual agent system – structured around a persona or character – that would offer users acceptable support and guidance based on self-management principles, acting as a focus for the user’s interactions. | Mental health issues related to having COPD. | Qualitative study | Adults with COPD felt that the supported self-management delivered by system was acceptable and engaging, particularly with regard to IOT capabilities. They felt the system would be particularly useful for individuals living alone. | / |
| Leveraging Natural Language Processing to Study Emotional Coherence in Psychotherapy | Use state-of-the-art language models for emotion recognition to automatically label clients’ utterance-level emotions during psychotherapy conversations, in order to examine the emotional coherence between verbally expressed emotions and self-reported emotions and whether emotional coherence would be associated with greater improvement in functioning throughout treatment. | 872 transcribed sessions from 68 clients in individual psychotherapy, not diagnosed as severely disturbed (due to a current crisis, past severe trauma or associated PTSD, a past or present psychotic or manic diagnosis, and/or current substance abuse). | Fine-tuned BERT-based language models and their corresponding lightweight adapter solutions: (a) XLM-RoBERTa; (b) HeBERT; (c) AlephBERT. | Comorbid anxiety and affective disorders, other comorbid disorders, anxiety disorders, affective disorders, relationship concerns, academic/occupational stress. | Exploratory and comparative study. | AlephBERT performed the best and achieved moderate accuracy in automatically labeling clients’ emotions at the utterance level. Findings indicated emotional coherence between verbally expressed emotions and self-reported emotions (both positive and negative). | / |
| Machine-Learning-Based Prediction of Client Distress From Session Recordings | Developed and evaluated NLP models that automatically predicted client symptoms of a given therapy session based on transcripts of their previous session. | 2,630 session recordings from 795 clients and 56 therapists. | RoBERTa as the primary representation of the text, using the base-English pretrained model. All models were trained using the Adam optimizer. In addition, an n-gram term frequency, inverse document frequency (TF-IDF) model with a regularized linear regression model has been employed. | The top 10 client-presenting concerns were anxiety, depression, academic performance, self-esteem, loneliness, social anxiety, relationship concerns with partner, family of origin, relationship concerns with friends, and body image. | Exploratory study. | The model was able to significantly predict client symptoms at an effect size similar to other psychotherapy process variables, yet do not relying on asking clients to rate sessions or therapists to guess about how a client is doing. Results highlight the potential for NLP models to be implemented in outcome-monitoring systems to improve quality of care. | Cohen’s d values between the first and last observed CCAPS ratings were –0.53 for overall distress, –0.34 for depression, –0.33 for generalized anxiety, –0.22 for social anxiety, –0.23 for academic distress, 0.11 for eating concerns, –0.24 for hostility, and –0.12 for substance use. |
| Integrating Bert With CNN and BiLSTM for Explainable Detection of Depression in Social Media Contents | 1) develop and evaluate fine-tuned BERT, BERT-BiLSTM, and BERT-CNN models, comparing their performance with MentalBERT; 2) observe the key features used by the BERT models to make the decision-making using TIBAV. | Depression Reddit Dataset, the Sentiment Analysis for Tweets Dataset, and the Mental Health Corpus. | Fine-tuned BERT, BERT-BiLSTM, and BERT-CNN. | Depression. | Exploratory and comparative study. | BERT-BiLSTM and BERT-CNN achieve superior performance compared to MentalBERT. BERT-CNN achieved exceptional accuracy scores of 0.982, 0.961, and 1.0 on the Depression Reddit Dataset, the Mental Health Corpus, and the Sentiment Analysis for Tweets Dataset, demonstrating its robust performance across different social media contexts. | / |
| Automated evaluation of psychotherapy skills using speech and language technologies | Demonstrate and analyze a platform able to process the raw recording of a psychotherapy session and provide, within short time, performance-based feedback according to therapeutic skills and behaviors expressed both at the utterance and at the session level. | Datasets drawn from large speech and language corpora both from the psychotherapy domain and from other fields, plus 5000 recordings drawn from its deployment in a real-world clinical setting used to assist training of new therapists. | Once the audio is recorded, it is automatically transcribed to find who spoke, when and what they said. If the transcription meets certain quality criteria, this textual information is used to predict utterance level and session-level behavior codes, which are summarized into an interactive feedback report.  The system gives comprehensive feedback to the therapist, including information about the dynamics of the session (e.g., talking time), low-level psychological language descriptors (e.g., type of questions asked), and other high-level behavioral constructs (e.g., understanding of the clients’ perspective). | Mental health issues. | Explorative study. | 4268 of the 5000 recordings met the quality criteria. | / |
| Predicting the language of depression from multivariate twitter data using a feature-rich hybrid deep learning model | Design and investigate the performance of a hybrid Deep Learning model for predicting and classifying users’ depressive sentiment from Twitter data. | Two datasets: D1) tweets from 2009 to 2016, 1402 depressed users and 292,564 tweets (labeled depressed only if user anchor tweet followed the strict pattern, “I was, I am, or I have been diagnosed depression”); D2) tweets from all users whose tweet do not contain the character string “depress” as of December 2016. | Hybrid CNN-LSTM Deep Learning model using TF-IDF, PCA, and Word2Vec approaches for classifying depressive sentiments, using significant linguistic features present within the text. | Depression. | Explorative and comparative study. | Combining CNN-LSTM andWord2Vec improved the performance of classification model by learning relationships between words by paying more attention to the most frequently used ones. The proposed model showed comparable performance with the hybrid deep learning-based models and outperformed state-of-the-art machine learning techniques with an accuracy of 96.78% and an MSE score of 3.21. |  |
| Using Topic Models to Identify Clients' Functioning Levels and Alliance Ruptures in Psychotherapy | Assess whether topic models could identify clients’ functioning levels and alliance ruptures, as well as examine whether and to what extent the topics identified would change over the course of treatment and whether this change was associated with treatment outcome. | Transcripts of 873 sessions from 58 clients treated by 52 therapists | The topic modeling process, starts by processing the input data through the analytic models used. The transcriptions were processed into documents that formed the input to the Topic model, which provided three outputs. The output in document-topic proportions was transformed to session-topic proportions. The vectors of these proportions were imported as X’s, along with the transcription labels as Y’s, to the sparse multinomial logistic regression model, which resulted in a prediction outcome and feature ranking. | The most common diagnoses were: comorbid anxiety and affective disorders, other comorbid disorders, anxiety disorders, affective disorders. | Exploratory study. | The model identified the labels above chance (65% to 75% accuracy). Change trajectories in topics were associated with change trajectories in outcome. The results suggest that topic models can exploit rich linguistic data within sessions to identify psychotherapy process and outcomes. | / |
| Just in time crisis response: suicide alert system for telemedicine psychotherapy settings | Design and validate a NLP machine learning model for detecting suicide content from patients-therapists psychotherapy transcripts to automatically identify and label the level of suicide risk and content expressed by the patient (i.e., risk factors, ideation, method, plan). | Transcripts of 1864 psychotherapy sessions (dyads) from a telemedicine platform (Talkspace). | NLP algorithm, based on a bag-of-words classification model, detecting and classifying suicidal content from patients’ expressions (first from vignettes, then from specific sentences). | Suicide. | Exploratory study. | The final NLP model identified risk-related content from non-risk content with good accuracy (AUC = 82.78). | / |
| Natural language processing of clinical mental health notes may add predictive value to existing suicide risk models | Evaluate whether REACH VET’s ability to predict death by suicide can be improved by including NLP-derived variables from unstructured EMR data. | Users that had been diagnosed with PTSD, between 2004 and 2013; at least PTSD diagnoses within 3 months (one in a mental health clinic). Final sample: 246 cases and 986 controls; total of 10244 notes selected for analysis. | Notes were analyzed using Sentiment Analysis and Cognition Engine (a Python-based NLP package). The output was evaluated using machine-learning algorithms. The area under the curve (AUC) was calculated to determine models’ predictive accuracy. | PTSD. | Exploratory study. | NLP derived variables offered small but significant predictive improvement (AUC=0.58) for patients that had longer treatment duration. | / |
